# Ocular Findings in Infants with Microcephaly Caused by Presumed Congenital Infection by Zika Virus in Sergipe

**DOI:** 10.1155/2020/7092432

**Published:** 2020-03-24

**Authors:** Marco Valadares, Anne Carolyne Lelis Oliveira Pedroso, Alessandro Santana, Angela Maria da Silva, Isabela Soares Costa, Maria Luiza Doria Almeida, Roque Pacheco de Almeida

**Affiliations:** Department of Medicine, Federal University of Sergipe, Sergipe 49060-100, Brazil

## Abstract

This study aims at identifying ocular findings in infants with microcephaly associated with presumed intrauterine infection by ZIKV. A cross-sectional study included 62 outpatient infants with congenital microcephaly, presumably secondary to maternal ZIKV infection. The included infants had head circumference below −2 standard deviations, with negative maternal serology for toxoplasmosis, rubella, cytomegalovirus, syphilis, and HIV. Assessment of ocular alterations was performed through review of their medical records. Forty two (67.7%) of the children analyzed presented some degree of ocular alteration. Findings in the posterior segment occurred in 29 (46.8%) patients, including atrophy of the retinal pigmentary epithelium in 15 (24.2%) patients, chorioretinal scars in 14 (22.6%) patients, retinal coloboma in 6 (9.7%) patients, and punctate retinal hemorrhage in 1 (1.6%) patient. Other ocular alterations were seen in 15 (24.2%) patients, including pathological strabismus in 11 (17.7%) patients, congenital cataracts in 2 (3.2%) patients, and nystagmus in 2 (3.2%) patients. Functional alterations were seen in four (6.5%) children. More than one change occurred in 11 (17.7%) children, eight of whom had head circumferences below −3 standard deviations. Changes in both the eyes occurred in 22 (35.5%) children, while 20 (32.3%) children had unilateral involvement. Among the 42 children with any ocular alteration, 27 (64.3%) children presented with severe microcephaly (head circumference with standard deviation lower than −3). The majority of children with microcephaly, presumably secondary to maternal ZIKV infection, present ocular alterations, with a higher frequency of involvement in the fundus. Severe ocular alterations are related to severe microcephaly.

## 1. Introduction

The Zika virus (ZIKV) is a flavivirus (family Flaviviridae) transmitted by the *Aedes aegypti* mosquito and was originally isolated from a feverish female Rhesus monkey in the Zika Forest in Uganda, in 1947 [1, 2]. It is closely related to other flaviviruses relevant to public health, such as the dengue (DENV), yellow fever (VFA), and the West Nile fever virus [3].

Cases of infection by ZIKV were confirmed for the first time in the northeast region (Bahia [4] and Rio Grande do Norte [5]) of Brazil, the first large country to experience the rapid spread of the ZIKV, at the beginning of 2015. Autochthonous cases of ZIKV have already been described in at least 22 states in the United States and 35 countries of the Americas [6].

The ZIKV epidemic was accompanied by an increased incidence of microcephaly in Brazil, reaching 1709 confirmed cases and 3182 cases still under investigation [7]. Congenital microcephaly may be accompanied by various alterations, and the most frequent being cognitive deficit, cerebral palsy, and epilepsy, in addition to behavioral disorder, dysphagia, and anomalies of the visual and auditory systems [8].

In January 2016, the first ophthalmologic findings in infants with microcephaly associated with ZIKV were published by researchers from the northeastern Brazilian state of Pernambuco: macular alterations (gross pigment mottling and/or chorioretinal atrophy) and changes in the optic nerve (hypoplasia with double-ring sign, pallor, and/or increased cup-to-disk ratio, iris coloboma, and subluxated lens) [9, 10]. More ocular alterations were subsequently reported, in addition to findings already mentioned by other authors in cities in the state of Bahia, [11] such as vascular tortuosity and subretinal hemorrhages [12].

Due to the recent and numerous association of ocular findings in infants with microcephaly associated with ZIKV, this article aims at identifying the changes most commonly found in children with microcephaly, which are presumably secondary to congenital infection by ZIKV, in the state of Sergipe.

## 2. Patients and Methods

A cross-sectional study was carried out in September 2016, by a medical chart review of 62 infants (37 females) with congenital microcephaly, who were followed at the University Hospital of Sergipe. This congenital condition was related to the presumed maternal infection by ZIKV.

The patients included were infants with circumference less than −2 standard deviations (SD) for respective age and sex (using the WHO curves as reference), with negative maternal serology for toxoplasmosis, rubella, cytomegalovirus, syphilis, and HIV.

A careful review of the medical records was performed by one examiner, who collected the ocular alteration statistics mentioned by the assisting ophthalmologist. The ophthalmologic monitoring of children was performed by one physician, through quarterly consultations, with the assessment of visual acuity (appropriate for the age of the infant) and examination of the anterior segment and fundoscopy in both the eyes. The findings were duly recorded in patient medical records.

The ophthalmologic evaluation was performed in this sequence: anamnesis with a detailed family and clinical history; mydriasis induced with tropicamide 1%, one drop repeated three times every 5 minutes; optical refraction test with retinoscope; anterior segment biomicroscopy in slit lamp; determination of the horizontal diameter of the cornea with millimeter ruler; measurement of intraocular pressure with the portable applanation tonometer; and indirect binocular ophthalmoscopy. Complementary tests were not performed, based on the findings of the clinical examination.

From circumference measurements, the children were classified with either nonsevere (SD between −2 and −3) or severe (SD less than −3) microcephaly, according to the Fenton curve.

The data found by the analysis of institutional medical records were tabulated in Excel and statistically analyzed according to simple and relative frequencies.

## 3. Results and Discussion

Table 1 shows the epidemiology of the group of 62 children studied. There is a predominance of female, full-term births, born by vaginal delivery and with adequate birth weight. The median, as well as the mode of the age, at the time of the examination was one month of life, with a minimum age of one month and a maximum of 8 months.

Alterations in vision and/or ocular architecture were found in 67.7% of the 62 children with microcephaly who were analyzed (Table 2).

Functional alterations, diagnosed through evaluation of visual behavior for the age group, were seen in 4 children (6.5%). Anterior segment changes were observed in only 2 patients (3.2%) with congenital cataracts. Other ocular alterations were pathological strabismus that was found in 11 (17.7%) patients and nystagmus in 2 (3.2%) patients.

**Figure 1:**
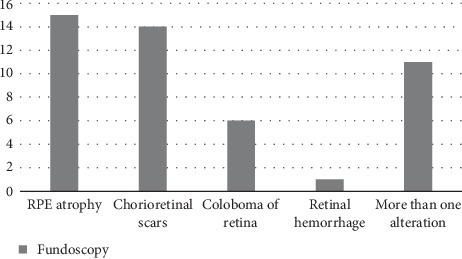
Frequency of ocular findings in the posterior segment of the eye through fundoscopy (in absolute numbers) in a sample of a microcephalic population at the University Hospital of the state of Sergipe, Brazil, in 2016.

Upon examination of the fundus, 29 patients (46.8%) presented alterations. The abnormalities found included retinal pigment epithelial atrophy in 15 (24.2%) patients, chorioretinal scars in 14 (22.6%) patients, retinal coloboma in 6 (9.7%) patients, and punctate retinal hemorrhage in 1 (1.6%) patients (Figure 1).

**Figure 2:**
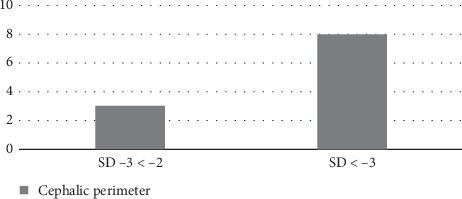
Frequency of more than one alteration in eye fundoscopy in relation to the measurement of cephalic circumference and consequent severity of microcephaly (in absolute numbers, in a sample of a microcephalic population at the University Hospital of the state of Sergipe, Brazil, in 2016).

Some patients had more than one alteration. These include 11 children (17.7%), eight of whom had cranial circumferences below −3 SD (Figure 2).

Bilateral ocular changes occurred in 12 patients (35.5%), while 20 patients (32.3%) presented only one eye involvement.

Of the 42 children with ocular alteration, 27 (64.3%) had severe microcephaly (head circumference with a standard deviation lower than −3).

## 4. Discussion

This study describes ocular alterations in 62 children with congenital microcephaly, which was presumably secondary to maternal infection by ZIKV. Specific ZIKV serology was not performed because positivity of the test available in Brazil is only expressed in the acute phase of infection [13]. The hypothesis of gestational infection by the virus was raised through exclusion of other causes of congenital microcephaly, together with the sudden increase of microcephaly cases in some states of Brazil after the recognized entry of ZIKV into the country.

Since the implementation of compulsory notification, at the beginning of 2016 until September of the same year, 261 cases of microcephaly were identified in the state of Sergipe. There were 13 deaths, of which six (6) were confirmed to be due to ZIKV, one (1) was excluded, and six (6) are pending investigation [14].

The rapid increase in the incidence of live births of children with microcephaly, after identification of the entry of the virus into Brazil, has gained the attention of doctors in the northeast of Brazil. Such doctors raised the initial hypotheses of the possible association of microcephaly with ZIKV infection during pregnancy [15].

A sudden increase in the number of cases of microcephaly in Brazil occurred in the second half of 2015, reaching 1709 confirmed cases in July of 2016 and 3182 cases remain under investigation. Of the total number of confirmed cases (1709), 267 were confirmed by laboratory criteria specific for the ZIKV. However, the Ministry of Health of Brazil considers that ZIKV infection occurs in the majority of mothers who had babies with final diagnoses of microcephaly. The proof of the microcephaly-ZIKV relationship is hampered by the low availability of tests for the laboratory diagnosis of ZIKV infection at the time of this study and in the convalescent phase of the disease [16]. The difficulty in confirming or excluding ZIKV infection still impairs our understanding of the natural history of the disease and its relationship with microcephaly [17].

Despite the difficulties in laboratory confirmations, the virus has already been proven to cross the placental barrier and reach the amniotic fluid and fetal tissues [18]. However, to date, it is not possible to state that the increase in microcephaly reports is exclusively related to the virus [19]. Cases with laboratory confirmation are few compared to the high number of notifications. Regardless, prevention measures are still required and need to be identified.

The inclusion criterion used in this study was the presence of microcephaly, with no infectious etiology as evidenced by agents classically described in the literature (TORCH and HIV). This is not a disease per se, but a sign of destruction or deficit of brain growth, and the definition by the World Health Organization is based on a head circumference of 31.9 cm or less for boys and 31.5 cm or less for girls born at full term, or below −2 SD (for sex and corresponding gestational age) for preterm births [4]. The sequelae of microcephaly will depend on its etiology and the age at which the event occurred. The earlier the disease occurs in the gestational period, the more serious is the central nervous system abnormalities [20]. In the case of ZIKV congenital syndrome, cerebral changes also appear to occur in the second and third trimesters of pregnancy [16]. The syndrome can occur with several alterations, including visual abnormalities [8].

There are a few published reports of ocular findings in ZIKV-related microcephaly. Ventura et al., in Pernambuco, observed visual disorders that included macular pigmentary changes (gross macular pigment mottling and/or macular neuroretinal atrophy), changes of the optic nerve (hypoplasia of the disk with a sign of double ring, pallor, and/or increase of papillary excavation), iris coloboma, and subluxated lens. The group stressed that changes of the retina are the most frequent [9, 10]. Miranda et al. in a study conducted in infants with microcephaly from Bahia reported a greater prevalence of alterations in retinal pigmentary epithelium and added findings of vascular tortuosities with a case of subretinal hemorrhages [12].

In agreement with the literature, this study showed a higher prevalence of alterations in the posterior segment of the eye (Table 1), with emphasis on retinal pigmentary atrophy. The varying eye fundoscopy findings (Figure 1) have also been reported in other studies [9, 10]. Strabismus was found in 11 patients, very frequent in the sample studied, and has not been described in the literature.

The clinical examination to evaluate the appropriate visual behavior for the age group described functional alterations in four children, in the absence of any ocular morphological changes. We cannot infer about visual acuity deficit due to limitations of this type of evaluation in the studied age group. The present study could not verify the cortical blindness hypothesis of these children. This condition needs to be confirmed through the visual evoked potential test, which did not exist in the hospital where the study was conducted.

de Paula Freitas et al. reported ocular findings in children with microcephaly in Salvador (Bahia) and highlighted that 70.0% of infants with such alterations presented bilaterally [11]. This study revealed bilateral ocular alterations in 52.4% of patients with ophthalmologic examination findings. The frequency of ocular alterations related to the degree of microcephaly has not been described in the literature. This study identified ophthalmologic findings in 42 children, of whom 64.3% presented with severe microcephaly. Furthermore, there are no reports in the literature that relate the severity of ocular alterations with the measurement of head circumference in children. The present study identified 11 patients with severe retinal findings (more than one type of change in eye fundoscopy), of whom eight (72.7%) patients had circumferences below −3 standard deviations for age and sex.

Prior to the ZIKV epidemic, microcephaly had already been related to ophthalmologic changes such as retinal atrophy and pigmentary retinopathy, diffuse lacunar chorioretinopathy, optic atrophy, optical hypoplasia, coloboma of optic nerve, nystagmus, cataract, retinal dysplasia, microphthalmia, microcornea, and vascular attenuation [12].

## 5. Conclusions

Brazil experienced a sudden increase in the number of cases of microcephaly in several states of the country, and there are strong indications that this is secondary to maternal infection by ZIKV. The confirmation of a greater number of cases of this association requires more accessible serologic tests that can contemplate the convalescent phase of infection.

In addition to an assessment of etiology, the approach of microcephaly repercussions (especially ocular consequences) requires greater attention. The knowledge of the possible alterations of this congenital syndrome will facilitate the monitoring of these children and enable better medical planning. Ultimately, a better understanding of ZIKV will help minimize inherent impairments and optimize possible interventions, particularly with respect to neurological and visual rehabilitation.

## Figures and Tables

**Table 1:** Epidemiological characterization of infants with microcephaly presumably caused by the Zika virus in Sergipe, Brazil, 2016.

Variable	*n*	%
Full-term	53	85.5
Female	35	56.5
Vaginal delivery	42	67.7
Absence of perinatal injury	62	100.0
Adequate for gestational age	42	67.7
Birth weight (g)^a^	2,755.5 ± 525.6	
Birth length (cm)^a^	45.9 ± 3.1	
Birth cephalic perimeter (cm)^a^	29.3 ± 2.1	

^a^Values are expressed as mean ± standard deviation (SD).

**Table 2:** Frequency of ocular alterations in a sample of the microcephalic population at the University Hospital of the state of Sergipe, Brazil, in 2016.

	Ocular alterations				
	Total number of infants	In the functional evaluation	In the anterior segment	In the posterior segment	Other ocular alterations
Absence	20	—	—	—	—
Presence	42	4	2	29	13

## Data Availability

The data used to support the findings of this study are available from the corresponding author upon request.
